# Diagnosis of a Right Congenital Diaphragmatic Hernia in a Neonate with POCUS

**DOI:** 10.24908/pocusj.v11i01.19486

**Published:** 2026-04-22

**Authors:** Alyssa DaVolio, Brandon S. Hays, Matthew O. Thompson, Michael J. Walsh, Parvesh M. Garg, Ricardo J. Rodriguez

**Affiliations:** 1 Atrium Health Wake Forest Baptist, Brenner Children's Hospital, Winston-Salem, NC, USA; 2 Department of Pediatrics, Neonatology, Wake Forest University School of Medicine, Winston-Salem, NC, USA; 3 Department of Pediatrics, Pediatric Cardiology, Neonatology, Wake Forest University School of Medicine, Winston-Salem, NC, USA; 4 Department of Pediatric Radiology, Wake Forest University School of Medicine, Winston-Salem, NC, USA

**Keywords:** POCUS, Congenital Diaphragmatic hernia, Point of Care Ultrasound, Neonatal Respiratory Distress

## Abstract

Point of care ultrasound (POCUS) can be used to diagnose neonatal lung diseases, especially in the setting of a newborn with significant respiratory distress. A neonate was born at 38.4 weeks gestational age to a 36-year-old Gravida 5 Para 3013 woman with prenatal concerns for fetal cardiothoracic anomalies. Fetal echocardiogram demonstrated left atrial compression of unclear etiology. After delivery, the neonate experienced significant respiratory distress. A transthoracic echocardiogram revealed pulmonary hypertension but no structural heart disease. A lung POCUS exam revealed a homogenous echogenic structure within the right pleural cavity consistent with liver parenchyma. The initial diagnosis of right Congenital Diaphragmatic Hernia (CDH) was made and confirmed by a radiology-performed lung ultrasound and a computed tomography (CT) scan of the chest. This case demonstrated the utility of lung POCUS as an initial diagnostic tool in the neonatal intensive care unit (NICU) for CDH.

## Introduction

Congenital Diaphragmatic Hernia (CDH) is a structural birth defect characterized by incomplete diaphragm development, which allows abdominal organs to herniate into the chest cavity [1]. CDH affects approximately 1 in 2,500 to 5,000 newborns, with a male-to-female predominance (1.5:1), and can result in significant morbidity and mortality [2–4]. There are multiple anatomic variants of herniation through the diaphragm, each causing a wide array of symptoms that typically relate to the degree of pulmonary hypoplasia and persistent pulmonary hypertension [1]. Antenatal diagnosis accounts for approximately 60% of all cases [5]. While left-sided CDH is more common overall, right-sided defects may cause symptoms of respiratory distress even within the first year of life [2].

Research into developing evidence-based guidelines for lung POCUS in the neonatal intensive care unit (NICU) has recently grown in popularity [6,7]. In neonates with respiratory distress, POCUS can be used to diagnose and assess the severity of different lung pathologies [6,9]. The benefits of lung POCUS in neonatal critical care include decreasing radiation exposure, quick and easy bedside availability, and repeatability for progression monitoring [7]. Using a high frequency linear transducer, a physician can quickly perform lung POCUS using the ribs, pleural lines, and diaphragm as landmarks to assess interactions between air and water on lung function and pathology [10,11]. Lung POCUS has been used for the diagnosis of a variety of conditions, such as congenital pulmonary airway malformations, meconium aspiration syndrome, pneumonia, transient tachypnea, respiratory distress syndrome, and pneumothorax [7,8]. Here, we present a case of a prenatally undiagnosed right CDH which was identified by lung POCUS in a newborn infant with signs of respiratory distress at birth.

## Case Presentation

A 36-year-old Gravida 5 Para 3013 woman presented to the Fetal Heart Program at Wake Forest University School of Medicine at 25 weeks gestational age due to concerns for a fetal cardiac anomaly. The maternal screens were unremarkable, and she had good prenatal care. The mother was counseled by pediatric cardiology and a fetal echocardiogram showed an abnormal contour of the left atrium, consistent with extrinsic atrial compression, and abnormal venous flow in the right hemithorax. The right pulmonary artery and right pulmonary veins were not well visualized. The right atrium and ventricle were notably dilated. Pediatric cardiology recommended an echocardiogram following birth to confirm findings. There was no recommended need for further imaging prenatally. The mother continued to be followed by the maternal-fetal medicine team throughout pregnancy. The maternal-fetal medicine team recommended the NICU team at the delivery of the infant with subsequent NICU admission for further work up.

**Figure 1.**
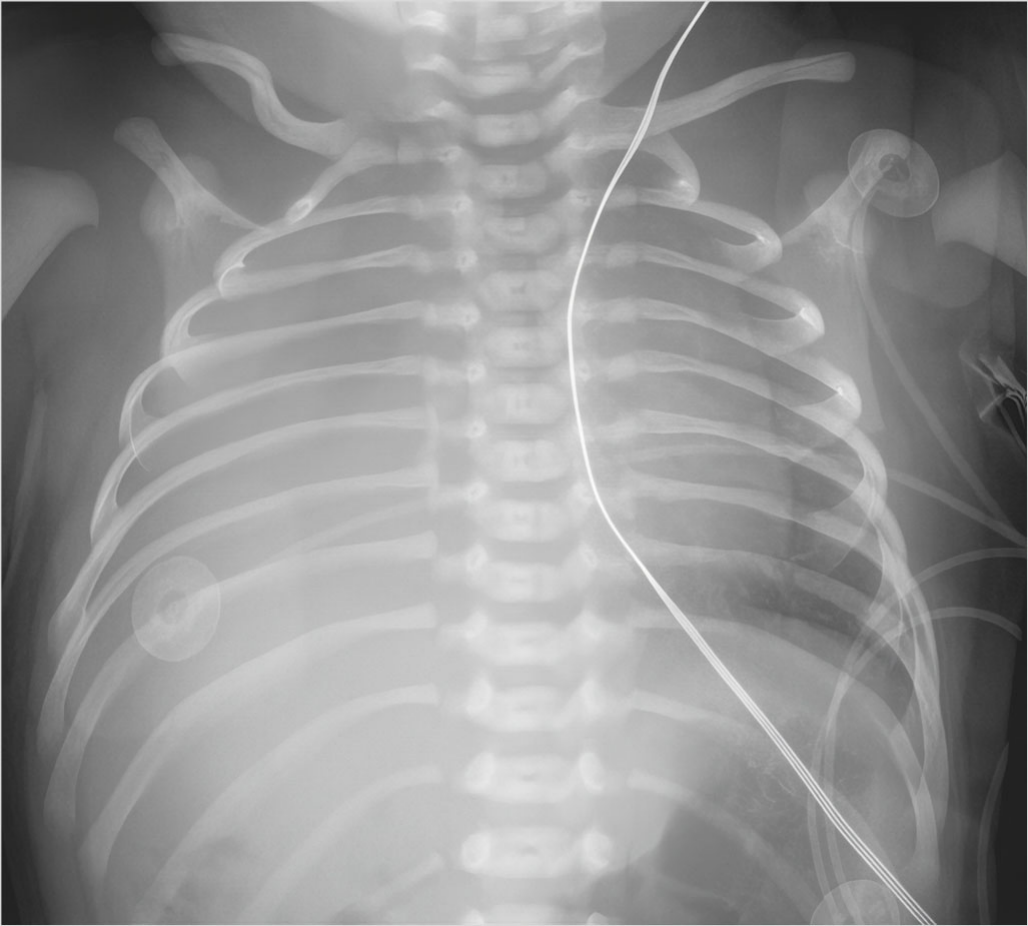
Single view chest X-ray performed on the first day of life. Complete opacification of the right chest is noted on chest film. The left chest shows a left-shifted heart border with decreased peripheral lung markings. Chest X-ray was read as “near-complete opacification of the right lung, favored atelectasis/collapse.”

The neonate was born at 38.4 weeks gestational age via repeat cesarean section. In the delivery room, the baby developed respiratory distress and required continuous positive airway pressure (CPAP) at +5 cm H_2_O with an FiO_2_ up to 70%. The neonate was admitted to the NICU on CPAP support, with FiO_2_ gradually weaned down to 21%. Chest X-ray on admission showed near-complete opacification of the right lung (Figure 1). A postnatal transthoracic echocardiogram performed by pediatric cardiology re-demonstrated external compression of the left atrium, dilation of the right atrium and right ventricle, and suprasystemic right ventricular pressure (90 mm Hg plus right atrial pressure, by tricuspid regurgitant jet). There was also improved visualization of right-sided pulmonary veins returning normally to the left atrium. However, the etiology of left atrium compression could not be elucidated.

**Figure 2.**
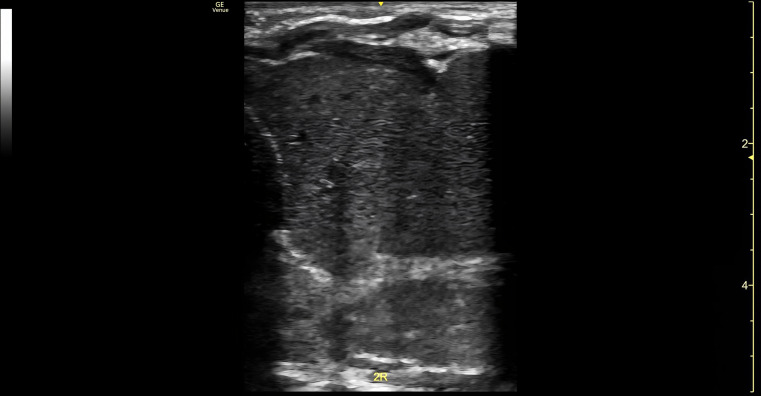
Point of care ultrasound (POCUS) of the right lung demonstrating homogenous echogenic structure within the right hemithorax consistent with liver parenchyma.

**Figure 3.**
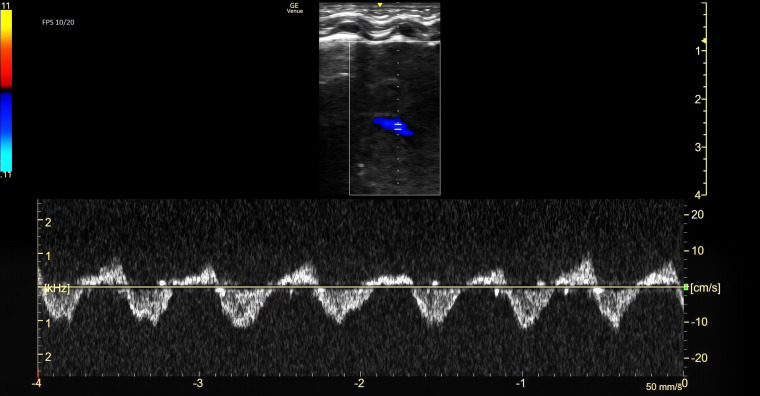
Point of care ultrasound (POCUS) of the right lung with color flow mapping and spectral Doppler demonstrating vascularity consistent with systemic venous flow.

**Figure 4.**
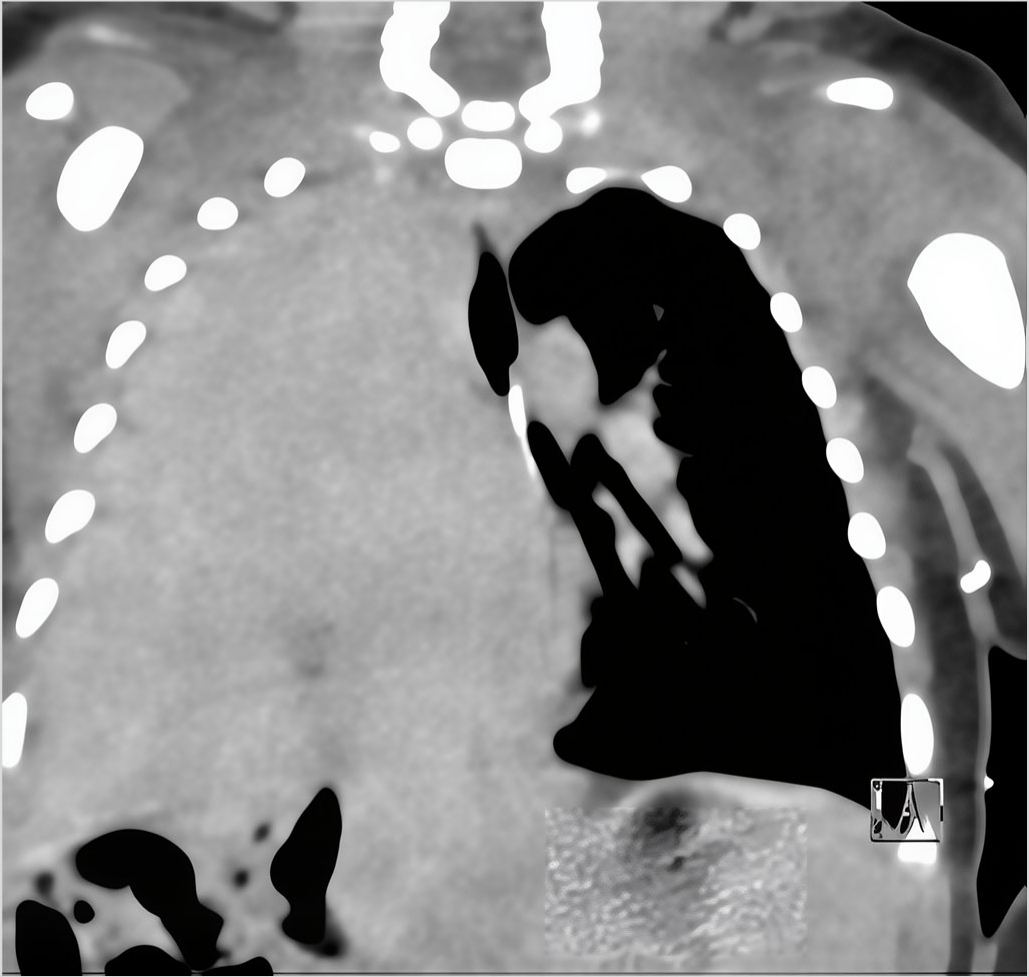
Computed tomography (CT) chest scan demonstrating a right diaphragmatic hernia with herniation of the liver into the right thoracic cavity.

Due to continued respiratory distress and chest X-ray findings of near-complete opacification of the right lung, a POCUS exam was performed. A Venue Go R2 Portable Ultrasound machine with a linear hockey-stick probe (GE HealthCare, USA) was used to obtain multiple lung views with the neonate lying supine. Both lungs were scanned in three zones: upper front, lower front and lateral for comparison. The left lung demonstrated normal lung sliding, with A lines and non-confluent B lines. The right upper lung zone showed lung sliding, presence of A lines and occasional B lines. The front lower and lateral zones of the right lung showed lack of lung sliding or A lines. Within the right hemithorax, a homogenous echogenic structure consistent with liver parenchyma was visualized extending into the abdomen (Figure 2). Color Doppler and spectral Doppler interrogation demonstrated the presence of blood flow within the structure consistent with portal venous flow (Figure 3). The characteristic hyperechoic line of the right hemidiaphragm was difficult to assess. No bowel loops or effusions were observed in the right hemithorax. These findings lead to the initial diagnosis of a right-sided congenital diaphragmatic hernia. A radiology-performed lung ultrasound was later completed with confirmed herniation of the liver into the right hemithorax. A CT of the chest was then obtained which confirmed the presence of a right-sided diaphragmatic hernia with herniation of the entire liver, a few bowel loops inferior to the hepatic margin, and the superior pole of the right kidney (Figure 4).

On the third day of life, the neonate was electively intubated in the operating room and underwent an exploratory laparotomy for CDH repair. Intraoperative findings included a large liver extending cephalad into the chest cavity with extensive hepato-pulmonary fusion—a finding not visualized on ultrasound or CT. Additionally, there was minimal diaphragm length posteriorly which impeded primary closure. The infant was returned to the NICU on mechanical ventilation with plans to revisit repair in 6–9 months. The immediate postoperative course was complicated by hemodynamic decompensation secondary to pulmonary arterial hypertension. The infant was treated with inhaled nitric oxide, low dose epinephrine, and milrinone infusions resulting in rapid improvement in mean arterial blood pressure and oxygenation index. The patient was eventually extubated and weaned to low flow nasal cannula with low inspired oxygen concentrations and received full enteral feeds by mouth and gastric tube (G-tube). The patient was ultimately discharged home without surgical interventions on full feeds and was then reportedly asymptomatic on room air and was thriving developmentally.

## Discussion

POCUS can help neonatal providers with the assessment of critical lung diagnoses, allowing for more prompt treatment and potentially improved outcomes [6]. Previous reports have described POCUS for the diagnosis of CDH in the pediatric emergency department; however, there is a dearth of literature on the use of POCUS for the initial diagnosis of CDH in the NICU [9,10]. This case demonstrates the utility of POCUS in the NICU to assess lung pathology in a symptomatic neonate.

The utility of lung POCUS for the diagnosis of effusions, pneumothorax, and pneumonia continues to be evaluated in literature. In a patient with respiratory distress and complete or near-complete hemithorax opacification, the diagnosis of these conditions can be rapidly performed with POCUS [12]. Furthermore, the evaluation of the diaphragm function may assist in ruling out extrapulmonary causes such as diaphragmatic eventration, paresis, or palsy [9]. In this case, the POCUS finding of liver parenchyma in the chest cavity, the lack of lung sliding, absence of A lines on the affected side in addition to the presence of systemic venous flow on spectral color mapping, and the lack of a clear delineation of the diaphragm anatomy led the medical team to the initial diagnosis of a right CDH.

POCUS can be useful as a rapid diagnostic tool for NICU providers to evaluate lung anatomy and pulmonary function, especially in the acutely decompensating patient who may not be stable enough for transport to the radiology suite [7]. Moreover, depending on local resources, a dedicated pediatric sonographer may not be readily available to perform a radiology-performed chest ultrasound on a 24-hour basis. Furthermore, the feasibility for sequential studies with no radiation exposure makes lung POCUS an attractive tool for monitoring disease progression, evaluating sudden changes in clinical status, and for rapidly implementing therapeutic maneuvers (e.g., chest tube placement and pericardiocentesis). Recently, Maddaloni et al. proposed a standardized POCUS protocol for the management of neonates with CDH [8]. These authors delineated a very comprehensive multifaceted protocol that included the use of advanced hemodynamic, abdominal, cerebral, lung, and diaphragm POCUS evaluations. However, POCUS has its own limitations—as demonstrated in this case by the inability to identify a hepato-pulmonary fusion. POCUS in the NICU is a growing practice which requires adequate training and understanding of the basic principles to apply diagnostic medical ultrasound. Thus, we cannot overemphasize the importance of continued collaboration with Pediatric Radiology and Pediatric Cardiology to maintain competence and quality assurance.

In summary, the utilization of lung POCUS led to early identification of a right-sided diaphragmatic hernia, which had not been antenatally visualized, and prompted further work-up to better assess and confirm the initial diagnosis. The early identification of the right-sided CDH allowed for collaboration between the medical and surgical teams in addition to changes in the approach to medical management.

## Conclusion

POCUS is a valuable tool for the rapid assessment of lung pathology in the symptomatic neonate. It may help providers implement appropriate and timely therapies and lead to improved outcomes.
